# Oxidative stress enhances tumorigenicity and stem-like features via the activation of the Wnt/β-catenin/MYC/Sox2 axis in ALK-positive anaplastic large-cell lymphoma

**DOI:** 10.1186/s12885-018-4300-2

**Published:** 2018-04-02

**Authors:** Chengsheng Wu, Nidhi Gupta, Yung-Hsing Huang, Hai-Feng Zhang, Abdulraheem Alshareef, Alexandra Chow, Raymond Lai

**Affiliations:** 1 Department of Laboratory Medicine and Pathology, University of Alberta, 5142J Katz Group Centre for Pharmacy and Health Research, Edmonton, AB T6G 1Z2 Canada; 2 Department of Oncology, University of Alberta, Edmonton, AB Canada; 3 DynaLIFEDX Medical Laboratories, Edmonton, AB Canada; 4 Department of Pathology and Laboratory Medicine, University of British Columbia, Vancouver, BC Canada; 5 Department of Laboratory Medicine and Pathology, Taibah University, Medina, Saudi Arabia; 6 Current Address: Department of Pathology, University of California San Diego, La Jolla, California USA

**Keywords:** Anaplastic large-cell lymphoma, Oxidative stress, Cancer cell plasticity, Intra-tumoral heterogeneity

## Abstract

**Background:**

The phenomenon that malignant cells can acquire stemness under specific stimuli, encompassed under the concept of cancer cell plasticity, has been well-described in epithelial malignancies. To our knowledge, cancer cell plasticity has not yet been described in hematopoietic cancers. To illustrate and study cancer cell plasticity in hematopoietic cancers, we employed an in-vitro experimental model of ALK-positive anaplastic large-cell lymphoma (ALK+ALCL) that is based on the phenotypic and functional dichotomy of these cells, with cells responsive to a Sox2 reporter (i.e. RR cells) being significantly more stem-like than those unresponsive to the reporter (i.e. RU cells).

**Methods:**

H_2_O_2_ was employed to trigger oxidative stress. GFP expression and luciferase activity, readouts of the Sox2 reporter activity, were quantified by using flow cytometry and luciferase activity assay, respectively. Doxorubicin-resistance and clonogenicity were assessed by using the MTS, methylcellulose colony formation and limiting dilution assays. Western blotting and quantitative PCR were used to assess the expression of various members of the Wnt/β-catenin pathway. Pull-down studies using a Sox2 binding consensus sequence were used to assess Sox2-DNA binding. Quercetin and 10074-G5 were used to inhibit β-catenin and MYC, respectively. siRNA was used to downregulate Sox2.

**Results:**

Under H_2_O_2_-induced oxidative stress, a substantial fraction of RU cells was found to convert to RR cells, as evidenced by their acquisition of GFP expression and luciferase activity. Compared to the native RU cells, converted RR cells had significantly higher levels of doxorubicin-resistance, clonogenicity and sphere formation. Converted RR cells were characterized by an upregulation of the Wnt/β-catenin/MYC/Sox2 signaling axis, previously found to be the key regulator of the RU/RR dichotomy in ALK+ALCL. Furthermore, Sox2 was found to bind to DNA efficiently in converted RR cells but not RU cells, and this finding correlated with significant elevations of several Sox2 downstream targets such as *WNT2B* and *BCL9*. Lastly, inhibition of β-catenin, MYC or Sox2 in RU cells significantly abrogated the H_2_O_2_-induced RU/RR conversion.

**Conclusions:**

We have demonstrated that cancer cell plasticity exists in ALK+ALCL, a type of hematopoietic cancer. In this cancer type, the Wnt/β-catenin/MYC/Sox2 axis is an important regulator of cancer cell plasticity.

**Electronic supplementary material:**

The online version of this article (10.1186/s12885-018-4300-2) contains supplementary material, which is available to authorized users.

## Background

The biological and clinical significance of intra-tumoral heterogeneity have been increasingly recognized [1]. Some degree of intra-tumoral heterogenicity is believed to be resulted from clonal evolution, a process in which individual cancer cell clones gradually acquire an increasing number of genetic defects and the subsequent selection of cell clones carrying highly aggressive or metastatic phenotype [2]. Intra-tumoral heterogeneity is also likely linked to the concept of cancer stem cells (CSCs), which represent a very small population within the tumors that are capable of self-renewal as well as generation of various elements of the tumors [1, 2]. In recent years, accumulating evidence has suggested that some of the bulk tumor cells can acquire cancer stemness under certain circumstances or specific stimulations [1–3]. This phenomenon is encompassed under the concept of “acquired stemness’ or cancer cell plasticity [4]. Thus far, cancer cell plasticity has been largely described and studied in epithelial and neurogenic malignancies. For instance, MCF7, an estrogen receptor-positive breast cancer cell line, was found to acquire higher levels of tumorigenic potential and chemoresistance upon oxidative stress [5]. In another study, it was found that hypoxic stress can result in the enrichment of CSCs in gliomas, which can be identified by means of their high CD133 expression [6]. To our knowledge, cancer cell plasticity has not been described in hematologic malignancies.

ALK-positive anaplastic large-cell lymphoma (ALK+ALCL) is a distinct form of T-cell non-Hodgkin lymphoma recognized in the World Health Organization Classification of Hematopoietic Neoplasms [7]. The majority of these tumors are found to carry the characteristic reciprocal chromosomal translocation involving *ALK* and *NPM*, and the resulted fusion gene product, *NPM-ALK* [7]. NPM-ALK has been shown to be the key oncogenic driver of ALK+ALCL [8]. A large body of experimental data has suggested that NPM-ALK mediates its oncogenic potential by exerting its constitutively active tyrosine kinase activity on a host of cellular signaling proteins, including those in the JAK/STAT, MAPK/ERK, and PI3K/AKT pathways, resulting in their inappropriate activation [8]. In our prior studies of ALK+ALCL, we had identified two distinct cell subsets in ALK+ALCL based on their differential response to a commercially available Sox2 reporter, which key components consist of the Sox2 regulatory region 2 (SRR2) as well as two gene reporters (*Green Fluorescence Protein, GFP* and *Firefly luciferase*) [9]. Specifically, we found that reporter responsive (RR) cells are significantly more tumorigenic and stem-like than reporter unresponsive (RU) cells. In a recently published manuscript, we also have reported that the Wnt/β-catenin/MYC/Sox2 signaling axis is the key regulator of the RU/RR dichotomy in ALK+ALCL, with this axis being substantially more active in RR cells than RU cells [10]. Due to the inclusion of the *GFP* gene in the Sox2 reporter, RU and RR cells present in ALK+ALCL cell lines can be easily detected and purified by using flow cytometry.

In this study, we found that a fraction of RU cells can convert to RR cells upon oxidative stress, and this conversion was associated with the acquisition of cancer stem-like features. We believe that these observations exemplify cancer cell plasticity in a hematologic malignancy. Our study results also have provided further support that the Wnt/β-catenin/MYC/Sox2 axis is the key regulator of cancer stemness in ALK+ALCL.

## Methods

### Cell lines and chemicals

Karpas 299, an ALK+ALCL cell lines, was a kind gift from Dr. Marshall Kadin (Boston, MA). SupM2, another ALK+ALCL cell lines, was purchased from Deutsche Sammlung von Mikrooorganismen und Zellkulturen GmbH (DSMZ), Germany (catalog number, ACC 509). Karpas 299 and SupM2 cell lines were grown in RPMI 1640 medium (Invitrogen, Life Technologies, Grand Island, NY) with supplementations of 10% fetal bovine serum (Invitrogen), 1% Penicillin/Streptomycin (ThermoFisher Scientific, Burlington, Ontario, Canada) and 2 μg/mL puromycin dihydrochloride (Sigma Aldrich, St Louis, MO) in 5% CO_2_ atmosphere at 37 °C. Chemicals used in this study, including hydrogen peroxide (H_2_O_2_), doxorubicin, N-acetyl-L-cysteine (NAC), iodonitrotetrazolium chloride, 10074-G5 and quercetin, were purchased from Sigma Aldrich.

### Flow cytometric isolation of RU and RR cells

RU and RR cells derived from SupM2 and Karpas 299 were purified with the use of a flow cytometry cell sorter (Becton Dickinson Biosciences, Franklin Lakes, NJ), and details of the method have been described previously [10].

### Luciferase reporter assay and flow cytometry

The luciferase reporter activity was measured by using the luciferase assay kit (Promega, Madison, WI), and the procedure was in accordance with the manufacturer’s protocol. The performance of flow cytometry has been detailed in a previous publication [9]. A software (FCS Express 5) from De Novo Software (Glendale, CA) was used to analyze the generated flow cytometry data.

### Trypan blue exclusion and MTS

Trypan blue exclusion and the MTS were used to quantify the number of viable cells (i.e. cell growth), and details of these assays have been previously described [11].

### Oxidative stress treatment

SupM2 or Karpas 299 cells growing in the log phase were seeded into 25 mL Corning™ cell culture flask (ThermoFisher Scientific Canada). For the H_2_O_2_ re-challenge experiments, cells were initially treated with two different doses of H_2_O_2_ (0.3 or 0.5 mM) for 3 days. On day 3, cells were subjected to centrifugation (50 X g, 10 min, room temperature), washed and cultured in fresh culture medium supplemented with either 0.3 mM or 0.5 mM H_2_O_2_ for two additional days (i.e. day 4 and day 5). At the end of the experiments, we performed luciferase reporter assay and flow cytometry to evaluate the luciferase activity and GFP expression, respectively. For the N-acetyl-L-cysteine (NAC) experiments, RU cells derived from SupM2 were treated with 0.3 mM H_2_O_2_ in the presence of 0–20 mM of NAC for 2 days, and these cells were assessed for their SRR2 reporter activity (i.e. luciferase activity and GFP expression). For the 10074-G5 or quercetin experiments, ALK+ALCL cells were treated with 5 μM of 10074-G5 or 50 μM of quercetin from day 4 to day 5 (24 h in total).

### Short interfering RNA and transfection

Short interfering RNA (siRNA, SMART pool, Dharmacon, Lafayette, CO) against Sox2 was used to down-regulate Sox2. Cells transfected with scrambled siRNA (Dharmacon) were included as the negative controls. BTX Electro Square ECM830 (225 V, 8.5 milliseconds, 3 pulses) was used to transfect siRNA species into ALK+ALCL cells. The efficiency of siRNA knockdown of Sox2 was assessed by using Western blotting.

### RNA extraction, cDNA synthesis, and quantitative reverse transcriptase PCR (qRT-PCR)

The method has been described previously [10]. Primer sequences used in this study were listed in Table 1.

**Table 1:** The primer sets for qRT-PCR performed in this study

Gene	Forward Primers	Reverse Primers
BCL9	5’-GGCCATACCCCTAAAGCACTC-3’	5’-CGGAAATACTTCGCTCCTTTT-3’
CTNNB1	5′- AAAGCGGCTGTTAGTCACTGG-3’	5′- CGAGTCATTGCATACTGTCCAT-3’
SOX2	5′- GCCGAGTGGAAACTTTTGTCG-3’	5′- GGCAGCGTGTACTTATCCTTCT-3’
WNT2B	5′- GATCAAGATGGTGCCAACTTC-3’	5′- CCAAGACACAGTAATCTGGAGAG-3’
GAPDH	5′- GGAGCGAGATCCCTCCAAAAT-3’	5′- GGCTGTTGTCATACTTCTCATGG-3’

### Western blotting

Details of the method have been described previously [11]. Antibodies reactive to phosphorylated ALK^Y1604^ (catalog #3341), ALK (D5F3®), phosphorylated STAT3^Y705^ (D3A7), STAT3 (124H6), phosphorylated MYC^S62^ (E1J4K), MYC (D84C12), Sox2 (D6D9), and β-catenin (D10A8) were purchased from Cell Signaling Technology (Danvers, MA). Antibody reactive to β-actin (sc-130300) was purchased from Santa Cruz (Dallas, TX), and antibody against active β-catenin (8E7) was from Merck Millipore (Toronto, ON, Canada). Anti-mouse IgG (catalog #7076) and anti-rabbit IgG (catalog #7074) were from Cell Signaling. Densitometry data was analyzed using Imag J software (National Institutes of Health, Bethesda, MD), and the values were normalized to those of β-actin or γ-tubulin.

### SRR2 probe binding assay

Details of this method have been described previously [10]. The sequence of the SRR2 probe is as follows:

5‘-AAGAATTTCCCGGGCTCGGGCAGC**CATTGTGATGCAT**ATAGGATTATTCACGTGGTAATG-3′. The underlined sequence is the SRR2 consensus sequence.

### Methylcellulose colony formation assay

The methylcellulose-based media were purchased from the R&D Systems Inc., (Minneapolis, MN). For each experimental group, we seeded 500 cells in each well, as described previously [11]. After ~ 10 days of culture, we employed iodonitrotetrazolium chloride to stain the colonies. Only colonies with > 40 cells were counted. Triplicate experiments were done. Images of the colonies were acquired using the Alphalmager HP (ThermoFisher Scientific Canada).

### Limiting dilution assay

ALK+ALCL cells, with or without H_2_O_2_ treatment, were seeded in RPMI1640 (Invitrogen) supplemented with 20% fetal bovine serum (Invitrogen), 1% penicillin/streptomycin and 2 μg/mL puromycin dihydrochloride (Sigma Aldrich) in 96-well low adherent plate (Corning). Ten different cell numbers, ranging from 1 to 1000 cells were used. After 7 days of culture, cells were stained with iodonitrotetrazolium chloride (Sigma Aldrich) for 24 to 48 h and images were acquired by using Alphalmager HP (ThermoFisher Scientific Canada). Cell spheres visible to naked eyes were counted. Triplicate experiments were used.

### Statistical analysis

The generated data in all quantitative assays of this study was presented as mean ± standard deviation. GraphPad Prism 5 software (La Jolla, CA) was employed for statistical analysis. The statistical significance of the differences between two experimental groups of test samples was assessed by using Student’s *t*-test. Statistical significance is denoted by * (*p* < 0.05) or ** (*p* < 0.01).

## Results

### Oxidative stress induces a conversion from RU to RR cells

**Fig. 1:**
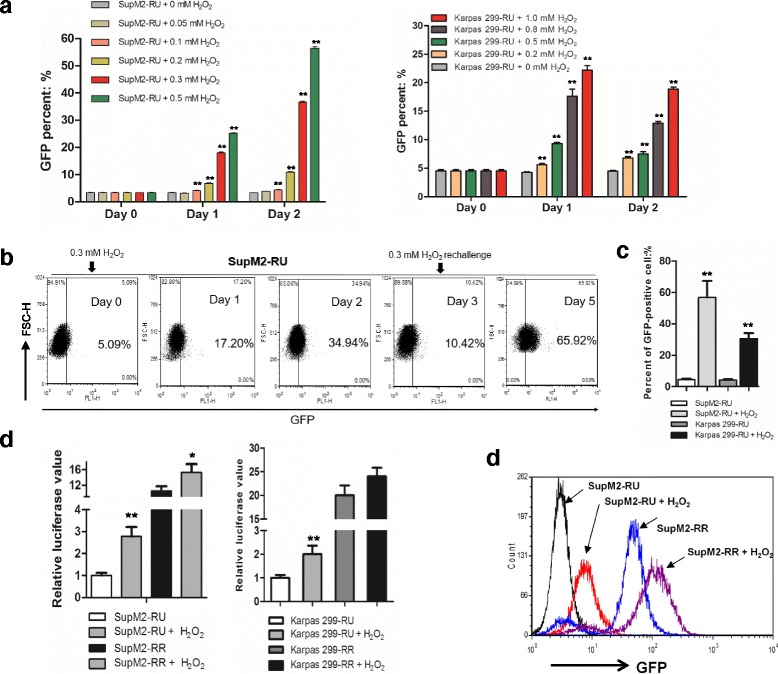
Oxidative stress induces the conversion of RU to RR cells. **a** RU cells derived from SupM2 and Karpas 299 cells were subjected to various doses of H_2_O_2_ for up to 2 days. The percent of GFP-positive cells was assessed by flow cytometry in RU cells from day 0 (the day of the treatment), day 1, and day 2. **b** The schematic experimental model of 0.3 mM H_2_O_2_ re-challenge treatment in RU cells derived from SupM2. **c** The GFP-positive cells in RU cells derived from SupM2 and Karpas 299 cells upon 0.3 mM and 0.5 mM H_2_O_2_ re-challenge treatment, respectively, at day 5. **d** The SRR2 luciferase activity in both RU and RR cells derived from SupM2 and Karpas 299 cells upon H_2_O_2_ re-challenge treatment at day 5. **e** The GFP-intensity of RU cells derived from SupM2, RU cells with H_2_O_2_ re-challenge, RR cells and RR cells with H_2_O_2_ re-challenge at day 5

In two of our recently published studies, we described that subsets of RU cells derived from esophageal squamous cell carcinoma and breast cancer cells can convert to RR cells when they were exposed to relatively low concentrations of H_2_O_2_, an agent known to generate reactive oxygen species (ROS) and induce potent oxidative stress [12, 13]. In this study, we asked if the H_2_O_2_-induced RU/RR conversion also exists in hematopoietic cancers such as ALK+ALCL. Two well-established ALK+ALCL cell lines, SupM2 and Karpas 299, were used. As shown in Fig. 1a, 2 days after H_2_O_2_ treatment (0–1.0 mM) of purified RU cells, GFP-positive cells became detectable by flow cytometry, with ~ 55% ‘converted RR’ cells at 0.5 mM of H_2_O_2_ for SupM2 and ~ 20% ‘converted RR’ cells at 1 mM of H_2_O_2_ for Karpas 299.

Since high doses of H_2_O_2_ are cytotoxic, we attempted to determine an optimal dose of H_2_O_2_ at which we can generate the highest number of viable converted RR cells for our studies. As shown in Additional file 1: Figure S1, we found this optimal H_2_O_2_ level to be 0.3 mM for SupM2, with which we generated ~ 30 viable converted RR on day 2, for ~ 70 RU cells used at the beginning of the experiment. For Karpas 299, this optimal dose was determined to be 0.5 mM H_2_O_2_, with which we generated ~ 40 viable converted RR cells on day 2, for ~ 100 RU cells used at the beginning of the experiment. To further increase the yield, we re-challenged ALK+ALCL cells with H_2_O_2_ on day 3. As shown in Fig. 1b-c, this experimental manipulation resulted in ~ 60% GFP-positive, converted RR cells derived from SupM2 and ~ 35% viable GFP-positive, converted RR cells derived from Karpas 299. This H_2_O_2_ stimulation protocol was used consistently for the remainder of this study. Correlating with these flow cytometry results, the luciferase activity in converted RR cells was significantly higher than that of native RU cells derived from both cell lines (Fig. 1d). Of note, native RR cells derived from SupM2 also showed significantly higher reporter activity (i.e. luciferase and GFP expression) upon H_2_O_2_ stimulation (Fig. 1d-e).

To confirm that the RU to RR conversion induced by H_2_O_2_ was directly linked to the cellular response induced by oxidative stress, we examined if the addition of N-acetyl-L-cysteine (NAC), a pharmacologic agent known to minimize cellular oxidative stress [14], can abrogate the conversion. As shown in Additional file 2: Figure S2A-B, in a dose-dependent manner, NAC significantly lowered the number of H_2_O_2_-induced GFP-positive cells as well as luciferase activity.

### Converted RR cells are phenotypically similar to native RR cells

**Fig. 2:**
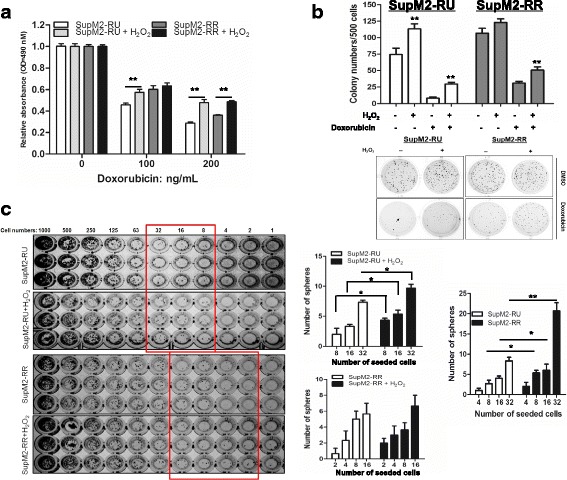
Converted RR cells share similar biological functions with native RR cells. **a** RU and RR cells from SupM2 cells were re-challenged with 0.3 mM H_2_O_2_ for 5 days. Then the cells were subjected to 0, 100, 200 ng/mL doxorubicin for 48 h. Cells without H_2_O_2_ treatment were included as control. **b** RU and RR cells derived from SupM2 cells were re-challenged with 0.3 mM H_2_O_2_ for 5 days. Then the cells were processed for methylcellulose colony formation assay in the presence/absence of 50 ng/mL doxorubicin, cells without H_2_O_2_ treatment were included as control. The graph demonstrated the number of colonies in the above experimental groups. The lower panel showed one representative result of triplicate experiments. Only colony with 40 cells (as pointed by the black arrow) or more was counted. **c** The serial diluted RU and RR cells derived from SupM2 were seeded in 96-well plate. After ~ 8 days, the number of spheres was counted in the highlighted wells circulated by the rectangle lines. The right panel showed the analyzed results which indicated that converted RR cells and native RR cells have formed more spheres in a lower number of cells seeded, in comparison with native RU cells

To address the question of whether converted RR cells are phenotypically similar to native RR cells, we compared these two types of cells with respect to chemo-resistance, clonogenicity and sphere-forming ability. First, to evaluate chemo-resistance, we subjected native RU and converted RR cells derived from SupM2 to various doses of doxorubicin, a chemotherapy drug commonly used to treat ALK+ALCL patients [7]. As shown in Fig. 2a, we found that converted RR cells exhibited significantly higher resistance to doxorubicin compared to native RU cells. Expectedly, RR cells stimulated with H_2_O_2_ were also significantly more resistant to doxorubicin than native RR cells at 200 ng/mL of doxorubicin.

Next we compared native RU and converted RR cells with respects to their clonogenicity by employing the methylcellulose colony formation assay. As shown in Fig. 2b, we found that converted RR cells derived from SupM2 formed significantly more colonies than native RU cells (113 ± 7 versus 74 ± 9, *p* = 0.008). This difference was observed even in the presence of doxorubicin. In comparison, when comparing native RR cells and RR cells stimulated with H_2_O_2_, we found that oxidative stress resulted in a significant increase in colony formation, but only in the presence of doxorubicin (Fig. 2b).

We then performed a comparison between native RU cells and converted RR cells regarding their sphere formation ability in a limiting dilution manner. As illustrated in Fig. 2c, we seeded increasing numbers of cells (i.e. 1 to 1000) in each of the wells in 96-well plates, and the lowest cell numbers in which colonies were visible by naked eyes were determined for native RU and converted RR cells. For SupM2, we found that a statistically significant difference between native RU and converted RR cells was found in 8 cells seeded in the wells. For Karpas 299, we found that a significant difference between native RU and converted RR cells was at 125 cells seeded in the wells (Additional file 3: Figure S3). We also compared native RR cells and RR cells stimulated with H_2_O_2_; a significant difference was found at 32 cells seeded in the wells for Karpas 299 cells (Fig. 2c and Additional file 3: Figure S3). The same experiment was performed using SupM2; no significant difference was observed.

Lastly, we assessed the growth rates of native RU cells, converted RR cells, native RR cells and RR cells stimulated with H_2_O_2_. As shown in Additional file 4: Figure S4, no significant differences were found. This result strongly argues against that the observed phenotypic differences between native RU and converted RR cells, as well as between native RR cells and H_2_O_2_-stimulated RR cells were simply due to a substantial difference in their growth rates.

### The Wnt/β-catenin/MYC/Sox2 axis is active in both converted RR cells and native RR cells

**Fig. 3:**
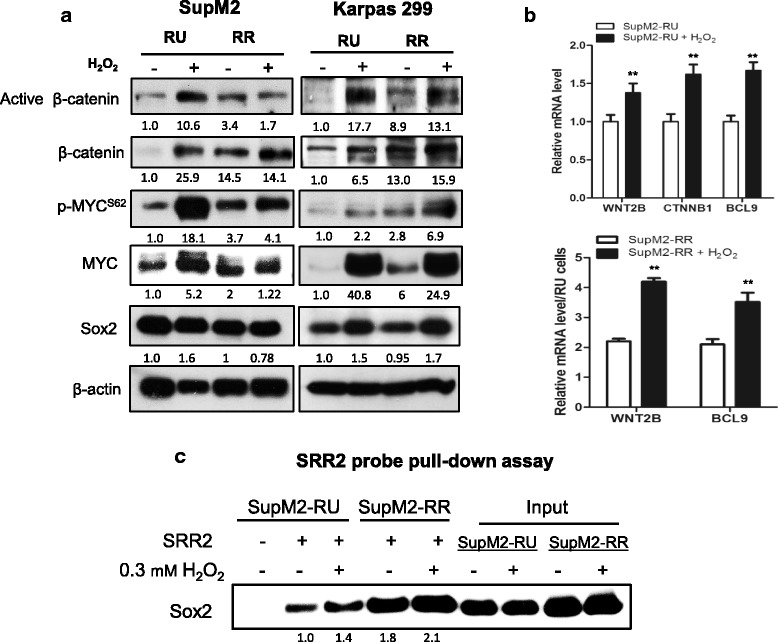
Converted RR cells share similar biochemical features with RR cells. **a** The Western blotting results showed the protein levels of active β-catenin, total β-catenin, p-MYC^S62^, and MYC in RU and RR cells derived from SupM2 and Karpas 299 cells, with or without H_2_O_2_ re-challenge. **b** The qRT-PCR results suggested that the Sox2 downstream targets including *WNT2B* and *BCL9* significantly increased in mRNA level in converted RR cells (upper panel) and RR cells re-challenged with H_2_O_2_ (lower panel), in comparison with their counterpart cells. **c** SRR2 probe pull-down assay was performed to evaluate the Sox2-SRR2 DNA binding ability. The results suggested that more Sox2 was pulled down by biotin-labeled SRR2 probe in converted RR cells and RR cells after H_2_O_2_ stimulation as compared to their native RU and RR cells. The input of the pull-down assay demonstrated that the Sox2 expression was not appreciably altered in this experiment. Imag J software was utilized to analyze the densitometry. Densitometry values of proteins of interest were all normalized to β-actin bands, and the densitometry value of protein of interest in RU cells was normalized as 1.0

We recently published that the Wnt/β-catenin/MYC/Sox2 axis is a key regulator of the RU/RR dichotomy [10]. Specifically, inhibition of this axis abrogated the RR phenotype and significantly decreased their clonogenicity and stem-like features [10]. In this study, we questioned if the H_2_O_2_-induced RU/RR conversion correlates with an activation of this signaling axis. As shown in Fig. 3a, compared to native RU cells, converted RR cells derived from SupM2 cells showed higher levels of active β-catenin, total β-catenin and p-MYC^S62^ (the active form of MYC) [15]. The same experiments were repeated using Karpas 299 cells and we found similar findings. In comparison, we did not identify any appreciable differences in the expression and activation of NPM-ALK or STAT3 between native RU and converted RR cells (Additional file 5: Figure S5).

To substantiate our finding that the Sox2 transcriptional activity was upregulated during the H_2_O_2_-induced RU/RR conversion, we employed quantitative real time-PCR (qRT-PCR) to assess the expression of several Sox2 downstream targets. As shown in Fig. 3b, the mRNA expression levels of *WNT2B* and *BCL9* were significantly higher in converted RR cells as compared to native RU cells (Fig. 3b). Similar results were observed when we compared native RR cells with RR cells stimulated with H_2_O_2_ (lower panel in Fig. 3b). Furthermore, we performed the Sox2-SRR2 probe binding assay. As shown Fig. 3c, substantially more Sox2 protein was pulled down by the biotin-labeled SRR2 probe in converted RR cells as compared to native RU cells. Furthermore, an appreciable increase of Sox2-SRR2 binding was identified in RR cells treated with H_2_O_2_ (Fig. 3c). Notably, the Sox2 protein level in both RU and RR cells derived from SupM2 was not appreciably changed in these experiments (Fig. 3c).

### Blockage of Wnt/β-catenin/MYC/Sox2 axis abrogates the RU to RR conversion

**Fig. 4:**
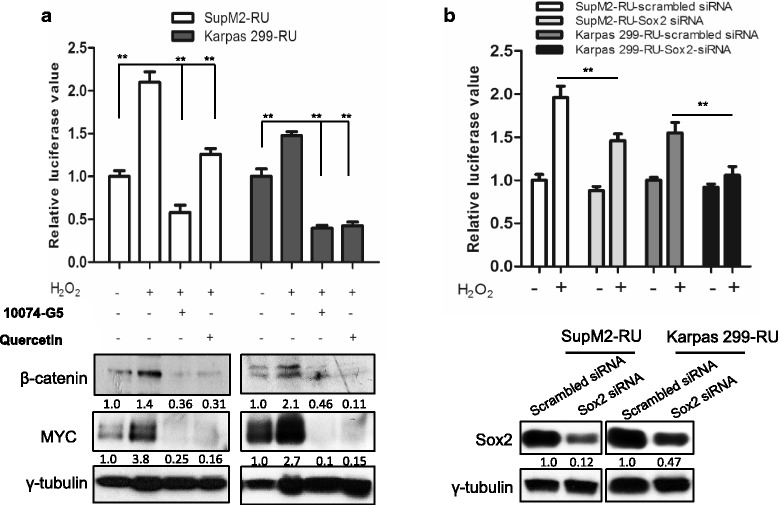
Blockage of Wnt/β-catenin/MYC/Sox2 axis abrogates the RU to RR conversion. **a** Pharmacological inhibition of MYC or β-catenin using 5 μM 10074-G5 or 50 μM quercetin for 24 h significantly decreased the SRR2 luciferase activities that were upregulated by H_2_O_2_ re-challenge. The Western blots below showed the knockdown efficiency of MYC and β-catenin after inhibitor treatment for 24 h. **b** RU cells with Sox2 knockdown by siRNA showed significantly decreased SRR2 luciferase activity in comparison to cells transfected with scrambled siRNA in the H_2_O_2_ re-challenge experiment. The Western blots below suggested the Sox2 knockdown efficiency at 48 h post siRNA transfection. Imag J software was used to analyze the densitometry. All proteins’ densitometry values were normalized to γ-tubulin bands, and the densitometry value of protein of interest in RU cells was normalized as 1.0

Next, we determined if the inhibition of the Wnt/β-catenin/MYC/Sox2 axis can significantly abrogate the RU/RR conversion induced by H_2_O_2_. To achieve this goal, we treated RU cells with quercetin (a β-catenin inhibitor) or 10074-G5 (a MYC inhibitor that can abrogate MYC-MAX heterodimerization and their DNA binding) [16] during the process of H_2_O_2_ challenge, as described in Methods and Materials. As shown in Fig. 4a, pharmacological inhibition of MYC or β-catenin dramatically decreased the expression level of MYC detectable by Western blots, and significantly reduced the luciferase activity in RU cells stimulated with H_2_O_2._ When we performed siRNA to knockdown Sox2 in RU cells from both ALK+ALCL cell lines, we found a significant decrease in the RU/RR conversion, as evidenced by the finding that the H_2_O_2_-induced increases in the luciferase activity were significantly attenuated (Fig. 4b). In addition, we found that siRNA knockdown of Sox2 in native RU cells derived from SupM2 also largely abrogated the H_2_O_2_-induced chemo-resistance to doxorubicin (Additional file 6: Figure S6)_._

## Discussion

In several recent reviews, the concepts and significance of cancer cell plasticity and acquired cancer stemness have been eloquently explained [2, 17, 18]. One of the key elements of these concepts is that the CSC is a dynamic phenotype, which can be acquired by non-stem cells if appropriate stimuli and microenvironment are provided. Virtually all of the published studies supporting these concepts are based on experimental data derived from malignant epithelial and neural-derived cells, and the induction of cancer stem-like features often involves the use of various chemotherapeutic agents, radiation therapy, hypoxia and oxidative stress [2, 17, 19, 20]. For instance, the size of the CSC population in head and neck squamous carcinoma cells, detectable by their ALDH^high^/CD44^high^ phenotype, was found to be significantly increased when these cells were exposed to cisplatin [21]. In two other studies, human pancreatic cancer cells subjected to a fibrogenic microenvironment were found to acquire CSC-like features [22, 23]. Hypoxia-inducible factors (HIFs), which exists in specific tumor microenvironment niche, is also known to promote metastasis, tumorigenicity and CSC-like features in certain human cancer models [24, 25].

The biochemical basis of how cancer cells acquire stemness is not completely understood, although there is evidence that activation of specific signaling pathways or transcription factors is likely important. In this regard, Saijo et al. have reported that oxidative stress-induced de-differentiation of lung cancer cells into CSCs is mediated via the activation of HOXA5 and upregulation of Sox2 [26]. In another study, hypoxia, which is also an inducer of oxidative stress [27], was found to increase the proportion of CD133-positive CSCs in glioma through the activation of the PI3K/AKT and ERK pathways [6]. A more recent study has shown that the tumor-propagating potential of glioblastoma cells can be enhanced by increasing the expression of a set of transcription factors normally involved in neuronal development, including *POU3F2*, *SOX2*, *SALL2* and *OLIG2* [28]. Epithelial-to-Mesenchymal Transition (EMT), which can be linked to the acquisition of cancer stemness [29, 30], is also known to be regulated by a number of cellular signaling pathways. For instance, activation of the RAS-MAPK pathway was found to induce EMT and increase the size of the CSC-like cell population [31]. Chaffer et al. have shown that non-CSCs derived from human breast cancers (CD44^low^) can convert to CSC’s (CD44^high^), and this conversion is mediated by ZEB1, an important EMT transcription factor [32]. The same group also demonstrated that TGFβ collaborates with the canonical and non-canonical Wnt signaling pathways to induce EMT [33].

Studies of acquired cancer stemness require appropriate experimental models. Most of the published studies in this field measured how various experimental manipulations change the size of the CSC populations, which are typically detected based on their expression of specific cell surface markers such as CD133 and CD44 [12, 20, 34–38]. In our studies, we employed a different approach. Specifically, the CSC-like population was defined, detected and isolated based on their responsiveness to a Sox2 reporter. In the literature, we are aware of a number of recent publications in which the CSC populations are also identified based on their differential responsiveness to the same Sox2 reporter used in this current study [13, 39–42]. In addition to the Sox2 reporter, we also found one recent publication in which the CSC population from colorectal cancer was identified based on their differential response to a Wnt reporter [38]. Similar to our RU/RR model, Wnt reporter responsive cells were found to be more stem-like than unresponsive cells, and a very small subset of reporter unresponsive cells were found to convert into reporter responsive cells upon stimulation with hepatocyte growth factor [38]. Taken together, we believe that the use of differential reporter responsiveness is a valuable experimental approach to study the acquisition of cancer stemness. In contrast with the use of cell surface markers, we believe that this experimental approach has certain advantages, as it provides direct clues to the biochemical mechanisms regulating the cancer stemness phenotype.

From our literature search, the concept of acquired cancer stemness has not yet been described in hematological malignancies, although a good number of CSC markers for these cancers have been proposed, including CD34+/CD38-/CD90−/interleukin 3 receptor(IL-3R)+/human leukocyte antigen (HLA)-DR/CD117- for acute myeloid leukemia (AML), CD34+/CD10-/CD19-/CD133+ for B-cell precursor acute lymphoblastic leukemia (ALL), and CD34+ for chronic myeloid leukemia (CML) [43, 44]. Regarding ALK+ALCL, we are aware of a publication in which tumor-propagating cells were identified based on their high Hoechst-efflux ability [45]. These tumor-propagating cells are featured with higher expression levels of NPM-ALK and ATP-binding cassette transporter G2 (ABCG2) as compared to bulk cell populations [45]. However, there was no evidence of a conversion of non-CSCs to CSCs in this study. Overall, we believe that we have demonstrated the first example of cancer cell plasticity in hematopoietic cancers.

We have previously demonstrated the central role of the Wnt/β-catenin/MYC/Sox2 axis as the defining feature of RR cells in ALK+ALCL [10]. Specifically, the high level of MYC permits the DNA binding of Sox2, and thus, promotes the transcription of Sox2 downstream target genes. One of the downstream target genes is *WNT2B*, which in turn upregulates the Wnt canonical pathway, resulting in an upregulation of MYC [10]. This positive feedback loop exists in RR cells but not RU cells [10]. In support of this model, we found that converted RR cells induced by H_2_O_2_ showed upregulation of this signaling axis. Compared to native RU cells, converted RR cells were found to have a substantially high expression level of Wnt2B, β-catenin, and MYC, as well as enhanced Sox2-SRR2 binding. In contrast, inhibition of β-catenin, MYC, or Sox2 resulted in significantly deceased conversion induced by oxidative stress. Although how exactly oxidative stress stimulates the Wnt/β-catenin/MYC/Sox2 axis is not known, redox oxidative species are known to directly activate the Wnt canonical pathway in HEK293 cells by stabilizing β-catenin and interrupting the interaction between dishevelled (Dvl) and nucleoredoxin (NRX), a strong inhibitor of Wnt/β-catenin signaling [46]. Further studies are required to determine if these mechanisms indeed underlie the RU to RR conversion in ALK+ALCL cells challenged by oxidative stress.

## Conclusions

Our study has demonstrated a novel experimental model in which the acquisition of CSC features can be induced by oxidative stress in ALK+ALCL, a hematologic malignancy. Our results have further substantiated the importance of the Wnt/β-catenin/MYC/Sox2 axis in conferring the cancer stem-like phenotype in ALK+ALCL.

## Additional files

Additional file 1: **Figure S1.** The cell numbers of RU cells derived from SupM2 and Karpas 299 upon various doses of H_2_O_2_ treatment were counted by trypan blue exclusion assay from day 0 to day 3. (PDF 79 kb)

Additional file 2: **Figure S2.** Anti-oxidant reagent NAC blocked the increase of GFP-positive cells induced by H_2_O_2_. A-B) Treatment of NAC abrogated the increased GFP-positive cells induced by 0.3 mM H_2_O_2_ for 48 h in RU cells derived from SupM2 in a NAC-dose dependent manner, read by GFP expression (A) and luciferase activity (B). (PDF 55 kb)

Additional file 3: **Figure S3.** The serial dilution experiment in RU and RR cells derived from Karpas 299 cells. The serial diluted RU and RR cells derived from Karpas 299 (from 1000 cells to 1 cell) were seeded in 96-well plates. After 8 days, the number of spheres was counted in the highlighted wells circulated by the rectangle lines. The right panel showed the analyzed results which indicated that converted RR cells and native RR cells with H_2_O_2_ stimulation have formed more spheres in a lower number of cells seeded (125 cells for RU cells and converted RR cells, 32 cells for RR cells and RR cells with H_2_O_2_ stimulation), as compared with native RU and RR cells, respectively. Note that RR cells also have formed more spheres than RU cells at a lower number of cells seeded (i.e. 32 and 63 cells). (PDF 259 kb)

Additional file 4: **Figure S4.** The cell growth of RU and RR upon H_2_O_2_ re-challenge. A-B) The cell growths of RU and RR cells derived from SupM2 and Karpas 299 after H_2_O_2_ re-challenge, assessed from day 1 (day 6 of H_2_O_2_ re-challenge experiment) to day 3. The results indicated that converted RR cells from both cell lines share similar cell growth rates with native RU cells, and RR cells after H_2_O_2_ re-challenge also grow in a similar rate with native RR cells. (PDF 103 kb)

Additional file 5: **Figure S5.** The activation levels of ALK and STAT3 were inappreciably changed upon H_2_O_2_ re-challenge. The expression levels of pALK^Y1604^, ALK, pSTAT3^Y705^, and STAT3 in RU and RR cells with or without H_2_O_2_ re-challenge. The same cell lysates from Fig. 3a were reused in this experiment, and note that the same β-actin blot as the one in Fig. 3a was recycled for H_2_O_2_-stimulation in RU and RR cells derived from Karpas 299 cells. (PDF 102 kb)

Additional file 6: **Figure S6.** RU cells derived from SupM2 were transfected with either Sox2 siRNA or scrambled siRNA which served as a negative control. Cells after siRNA transfection were exposed to 0.3 mM H_2_O_2_ re-challenge. At day 4 of the H_2_O_2_ re-challenge experiment; cells were subjected to 200 ng/mL doxorubicin for additional 48 h, following by the trypan blue exclusion assay-based cell viability analysis. The Western blots in the right panel demonstrated the Sox2 knockdown efficiency in RU cells from SupM2 24 h post transfection. (PDF 48 kb)

## Abbreviations

ALK+ALCL: ALK-positive anaplastic large-cell lymphoma
CSCs: cancer stem cells
GFP: Green Fluorescence Protein
H_2_O_2_: hydrogen peroxide
NAC: N-acetyl-L-cysteine
qRT-PCR: quantitative real-time polymerase chain reaction
RR: reporter responsiveness
RU: reporter unresponsiveness
siRNA: short interfering RNA
