# Supplementary figures and images for: Oxidative stress enhances tumorigenicity and stem-like features via the activation of the Wnt/β-catenin/MYC/Sox2 axis in ALK-positive anaplastic large-cell lymphoma
Source: BMC Cancer. 2018 Apr 2;18:361. doi: 10.1186/s12885-018-4300-2 (PMC5879562; doi:10.1186/s12885-018-4300-2)

Additional file 1 figure S1

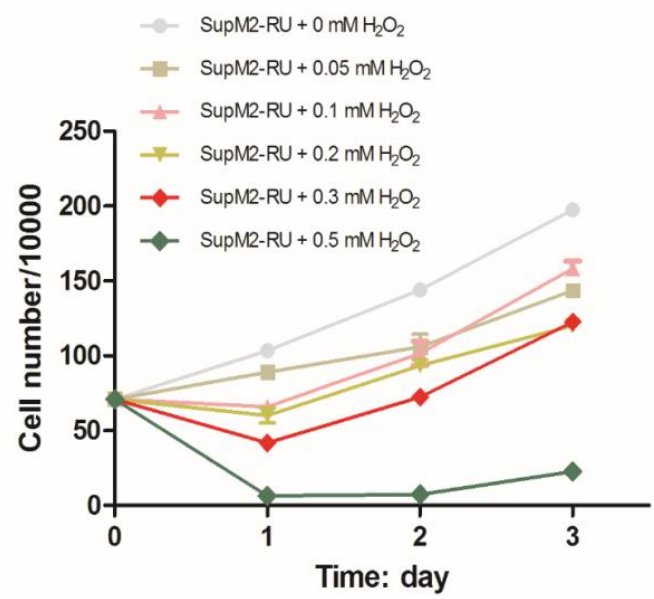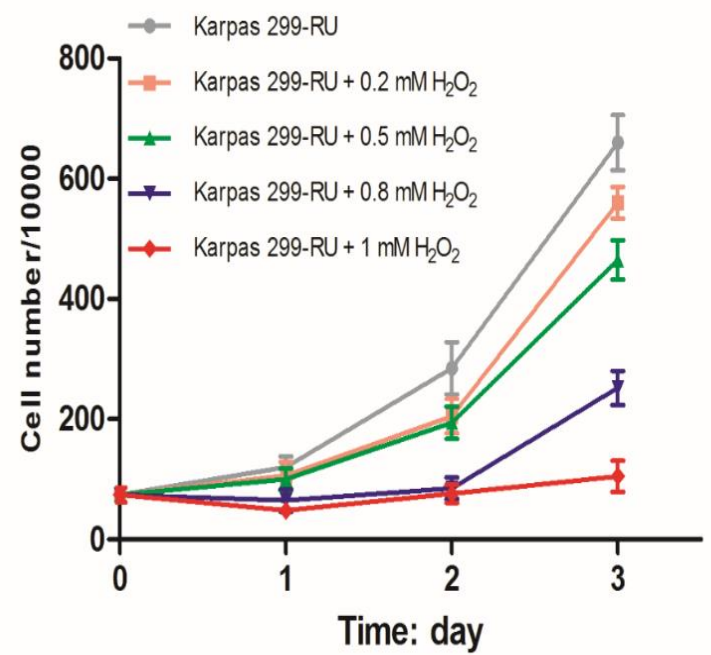

Supplement: Supplementary file 1 — Figure S1. The cell numbers of RU cells derived from SupM2 and Karpas 299 upon various doses of H2O2 treatment were counted by trypan blue exclusion assay from day 0 to day 3. (PDF 79 kb) [file 12885_2018_4300_MOESM1_ESM.pdf]

**A**

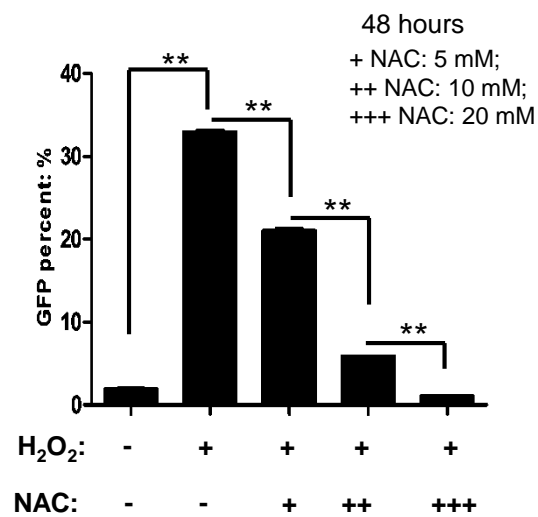

**B**

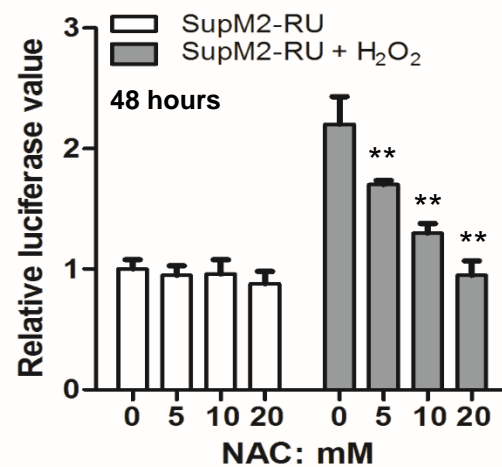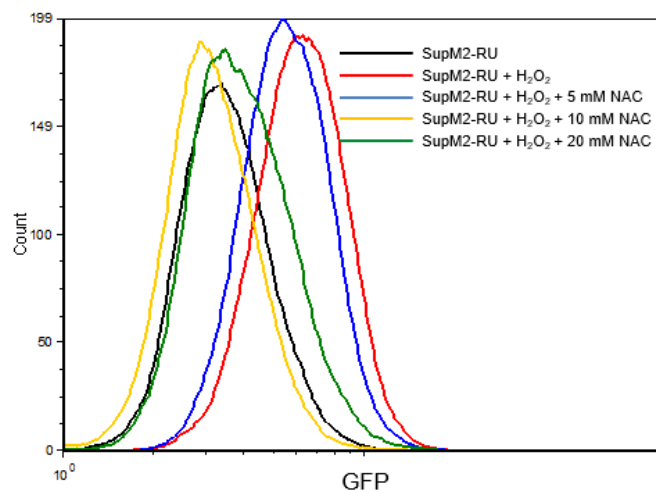

Supplement: Supplementary file 2 — Figure S2. Anti-oxidant reagent NAC blocked the increase of GFP-positive cells induced by H2O2. A-B) Treatment of NAC abrogated the increased GFP-positive cells induced by 0.3 mM H2O2 for 48 h in RU cells derived from SupM2 in a NAC-dose dependent manner, read by GFP expression (A) and luciferase activity (B). (PDF 55 kb) [file 12885_2018_4300_MOESM2_ESM.pdf]

Additional file 3 figure S3

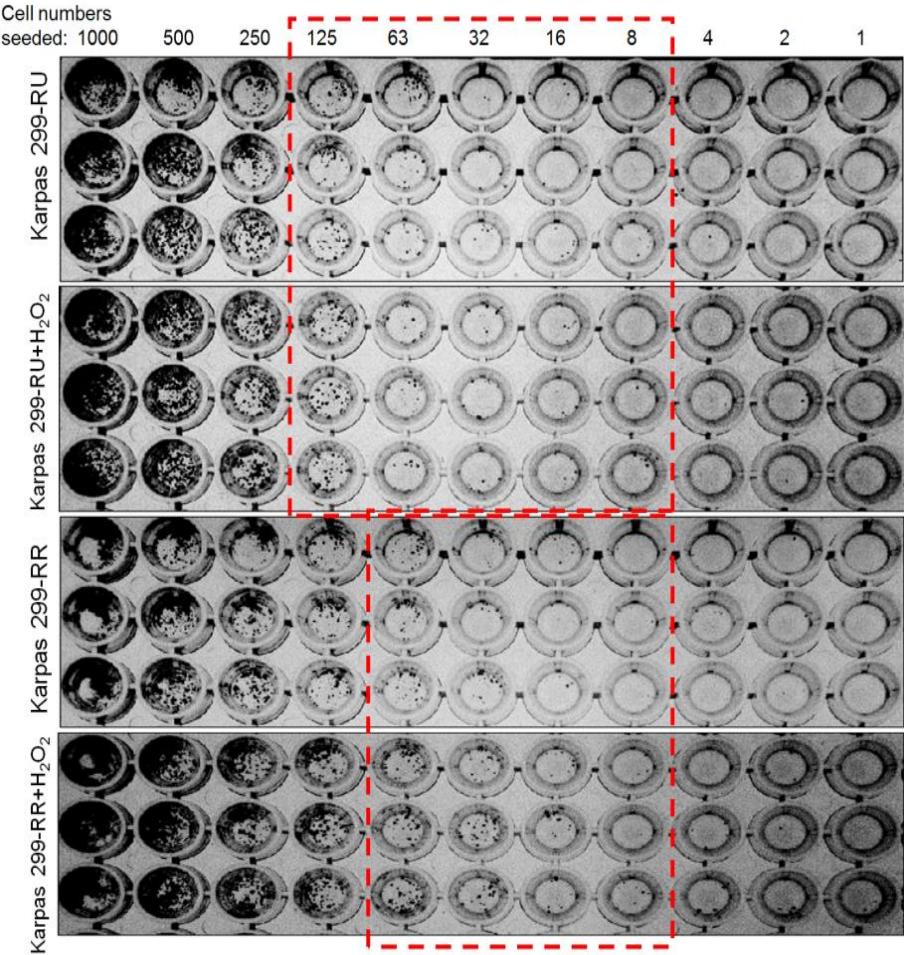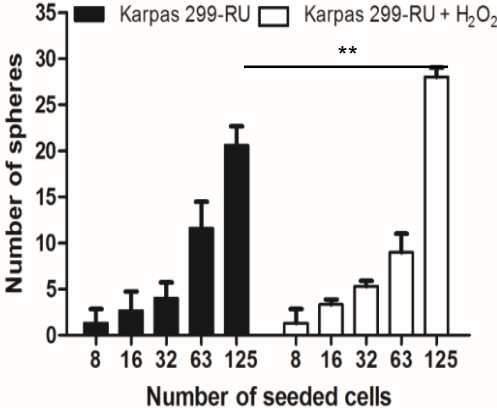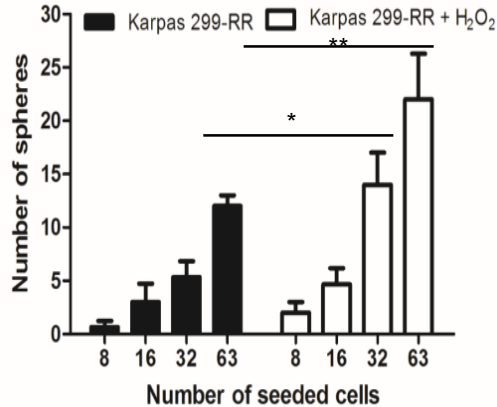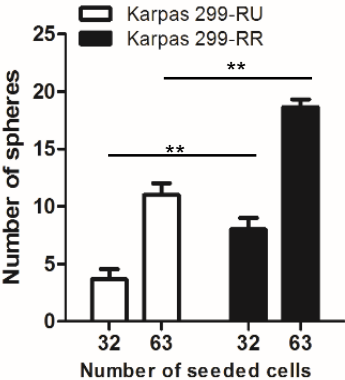

Supplement: Supplementary file 3 — Figure S3. The serial dilution experiment in RU and RR cells derived from Karpas 299 cells. The serial diluted RU and RR cells derived from Karpas 299 (from 1000 cells to 1 cell) were seeded in 96-well plates. After 8 days, the number of spheres was counted in the highlighted wells circulated by the rectangle lines. The right panel showed the analyzed results which indicated that converted RR cells and native RR cells with H2O2 stimulation have formed more spheres in a lower number of cells seeded (125 cells for RU cells and converted RR cells, 32 cells for RR cells and RR cells with H2O2 stimulation), as compared with native RU and RR cells, respectively. Note that RR cells also have formed more spheres than RU cells at a lower number of cells seeded (i.e. 32 and 63 cells). (PDF 259 kb) [file 12885_2018_4300_MOESM3_ESM.pdf]

Additional file 4 figure S4

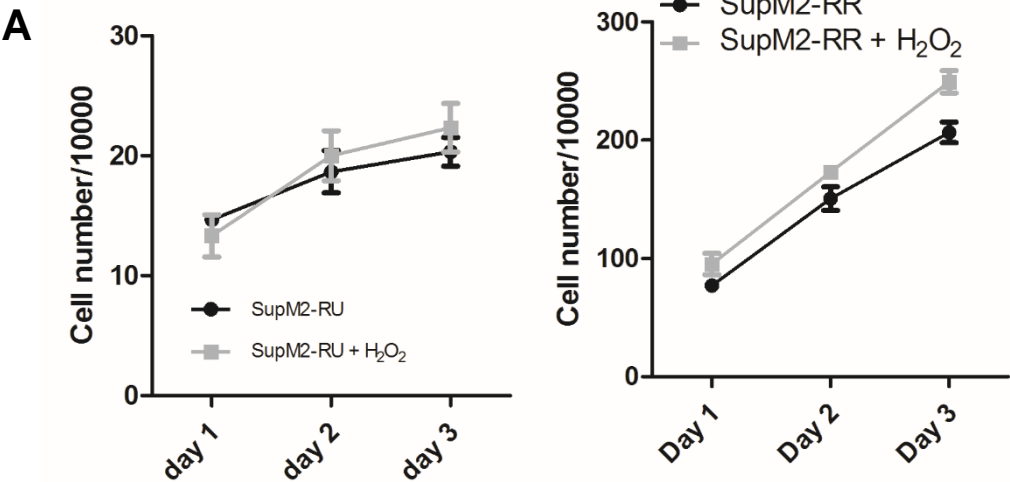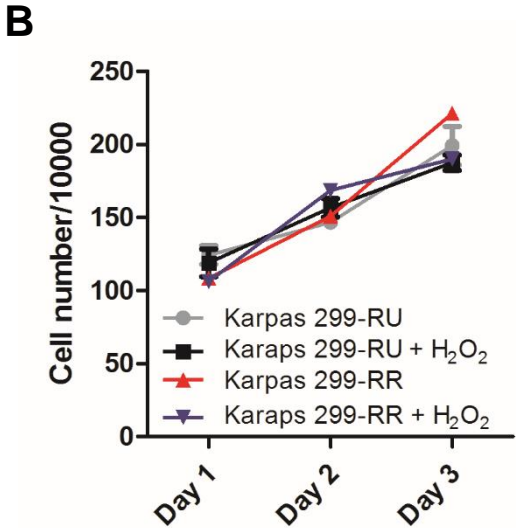

Supplement: Supplementary file 4 — Figure S4. The cell growth of RU and RR upon H2O2 re-challenge. A-B) The cell growths of RU and RR cells derived from SupM2 and Karpas 299 after H2O2 re-challenge, assessed from day 1 (day 6 of H2O2 re-challenge experiment) to day 3. The results indicated that converted RR cells from both cell lines share similar cell growth rates with native RU cells, and RR cells after H2O2 re-challenge also grow in a similar rate with native RR cells. (PDF 103 kb) [file 12885_2018_4300_MOESM4_ESM.pdf]

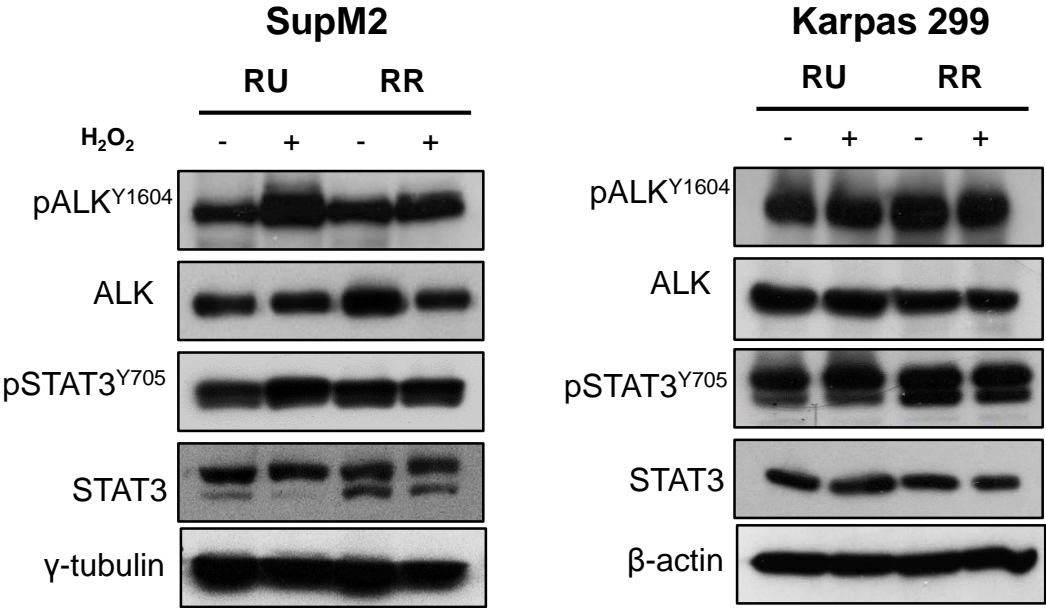

Supplement: Supplementary file 5 — Figure S5. The activation levels of ALK and STAT3 were inappreciably changed upon H2O2 re-challenge. The expression levels of pALKY1604, ALK, pSTAT3Y705, and STAT3 in RU and RR cells with or without H2O2 re-challenge. The same cell lysates from Fig. 3a were reused in this experiment, and note that the same β-actin blot as the one in Fig. 3a was recycled for H2O2-stimulation in RU and RR cells derived from Karpas 299 cells. (PDF 102 kb) [file 12885_2018_4300_MOESM5_ESM.pdf]

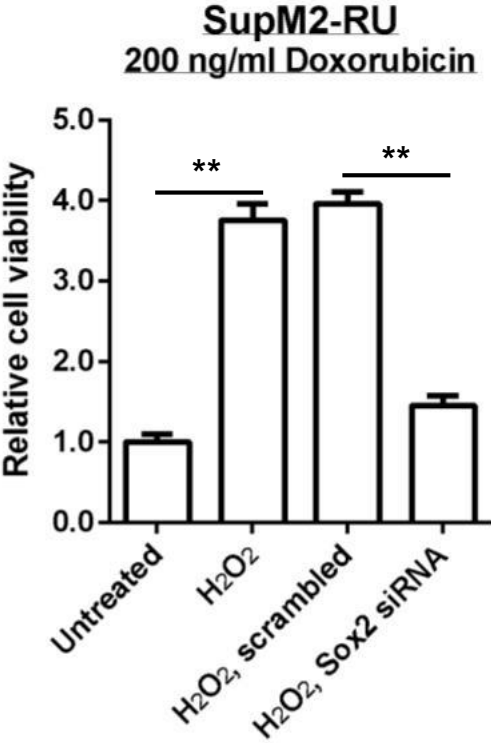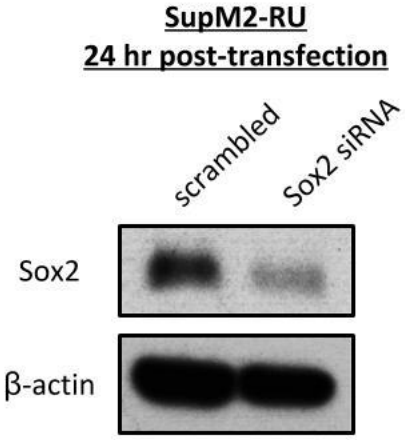

Supplement: Supplementary file 6 — Figure S6. RU cells derived from SupM2 were transfected with either Sox2 siRNA or scrambled siRNA which served as a negative control. Cells after siRNA transfection were exposed to 0.3 mM H2O2 re-challenge. At day 4 of the H2O2 re-challenge experiment; cells were subjected to 200 ng/mL doxorubicin for additional 48 h, following by the trypan blue exclusion assay-based cell viability analysis. The Western blots in the right panel demonstrated the Sox2 knockdown efficiency in RU cells from SupM2 24 h post transfection. (PDF 48 kb) [file 12885_2018_4300_MOESM6_ESM.pdf]
